# Exploring Limits of [1‐^13^C]Pyruvate Nuclear Spin Relaxation: Impact of Field, Deuteration, Degassing, and Additives

**DOI:** 10.1002/smsc.70391

**Published:** 2026-08-30

**Authors:** Josh P. Peters, Jan‐Bernd Hövener, Andrey N. Pravdivtsev

**Affiliations:** ^1^ Section Biomedical Imaging (SBMI), Molecular Imaging North Competence Center (MOIN CC), Department of Radiology and Neuroradiology, University Hospital Schleswig‐Holstein, Kiel University Kiel Germany

**Keywords:** hyperpolarization, magnetic field cycling, nuclear magnetic resonance, pyruvate, relaxation

## Abstract

Limited lifetime is the most prominent hurdle for hyperpolarized magnetic resonance imaging. Today, [1‐^13^C]pyruvate is the most widely used metabolic hyperpolarized probe, but low signal‐to‐noise ratio (SNR) for metabolites such as lactate remains a critical challenge. As post‐administration relaxation is difficult to affect, reducing polarization losses during delivery, purification, and administration is key for increasing SNR. To elucidate the relaxation mechanisms governing [1‐^13^C]pyruvate *T*_1_, we evaluate 35 sample compositions across magnetic fields from a few µT to 9.4 T, varying solvent and agent deuteration, buffer type and concentration, degassing, and additives. Field‐dependent *T*_1_ profiles reveal distinct mechanistic contributions from each factor, with degassing and deuteration exerting the strongest effects at low fields relevant to clinical transfer conditions. Notably, buffer composition has an unexpected and substantial impact on *T*_1_, a finding largely overlooked in prior literature. Under optimized conditions, [1‐^13^C]pyruvate *T*_1_ at Earth’s field is extended by almost 8 times, from ~30 to ~230 s. This reduces polarization losses after a 20–60 s transfer interval by 3.6–5.6‐fold, thus increasing SNR. These findings are also important for hyperpolarized probes beyond pyruvate, which exhibit faster low‐field relaxation and stand to benefit even more substantially from systematic control of sample composition.

## Graphical abstract

The lifetime of hyperpolarized [1‐^13^C]pyruvate is governed by its chemical surroundings. Systematic mapping of 35 sample compositions across magnetic fields from a few µT to 9.4 T identifies solvent deuteration, degassing, buffer composition, and storage field as independent handles on relaxation. Together, they extend *T*_1_ fourfold, turning sample preparation from trial and error into rational design.

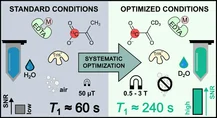

## 1 Introduction

The hyperpolarization of nuclear spins (HP) allows for increasing the nuclear magnetic resonance (NMR) and magnetic resonance imaging (MRI) signals of selected probes by multiple orders of magnitude compared to thermal equilibrium. However, HP is a transient state with a characteristic relaxation rate, which depends on many factors. As a result, there is often a strong signal from the injected agent at the beginning, and a rather low one for the later‐appearing metabolites. Overall, the signal‐to‐noise ratio (SNR) of hyperpolarized MRI in vivo is rather poor, reflected in low spatial resolution and short observation windows.

The SNR of the metabolites of interest (MOI) critically depends on the polarization at the time of metabolism (*P*_ATOM_) and other factors, such as concentration. The polarization before metabolism (*P*_BM_) is, in turn, determined by the polarization upon leaving the polarizer and by its subsequent relaxation, first in vitro and then in vivo (after administration). The polarization level can be affected, e.g., by improved HP techniques and equipment. The polarization relaxation of relevant HP probes is an essential metric in most works; quantitative impacts of varying sample compositions across orders of magnetic field strengths, however, have rarely been discussed.

Indeed, for the most commonly used HP probe [1‐^13^C]pyruvate, the time for purification and transfer (*t*_pt_) is often around 1 min [1, 2, 3, 4, 5] ― which is of the order of *T*_1_: only 100% (exp[‐*t*_pt_/T_1_]) ~37% of the initial polarization is retained. Hence, to increase the *P*_ATOM_, there are three options: increase initial polarization (which is over 50% for pyruvate, already close to the practical limit), shorten purification and transfer, decelerate relaxation, or a combination of all. Although dissolution dynamic nuclear polarization is the only technology used in clinical settings to date [1, 6, 7], other hyperpolarization methods—particularly parahydrogen‐induced polarization by side‐arm hydrogenation (PHIP‐SAH) and signal amplification by reversible exchange (SABRE)—are rapidly progressing from proof‐of‐principle to in vitro and in vivo applications, with an increasing portfolio of efficiently polarizable metabolites [8, 9, 10, 11, 12, 13].

For some molecules, for example, nicotinamide [14, 15], succinate [16, 17, 18], and fumarate [18], at certain pH and low magnetic fields, all hyperpolarization can be lost during the transfer. Some additives, such as ethylenediaminetetraacetic acid (EDTA), which chelate metal cation impurities, extend HP lifetime [19]. In our previous work, we described the effect of chemically induced deceleration of nuclear relaxation (CIDER) [14, 15], in which various additives were shown to reduce *T*_1_ (spin‐lattice) relaxation of some (not all) ^15^N nuclei. To do so, we used hydrogen bonding additives such as nicotinamide, urea, glycerol, or dendrons.

We applied CIDER‐like optimization to the most hyperpolarized probe, [1‐^13^C]pyruvate, and achieved over 4 min of *T*_1_ [19]; however, a comprehensive analysis of pyruvate relaxation as a function of field, chemicals, preparation, and combinations is still lacking. While the shortening of *T*_1_ by radicals and the scavenging of radicals were examined [20, 21], the impact of other chemicals like buffer, its type, and concentration, or salts was not. Often, deuteration decelerates relaxation [22, 23, 24, 25]; however, the previous use of deuterated pyruvate [26] or even utilization of so‐called long‐lived spin states in [1,2‐^13^C_2_]pyruvate [27, 28] did not change the relaxation loss radically.

Quantifying relaxation at low fields is not straightforward. Magnetic field cycling (MFC) techniques, which are indispensable for understanding molecular and spin properties [29, 30, 31, 32, 33, 34, 35], have been used to estimate polarization losses for hyperpolarized pyruvate [30, 33, 35].

**FIGURE 1:**
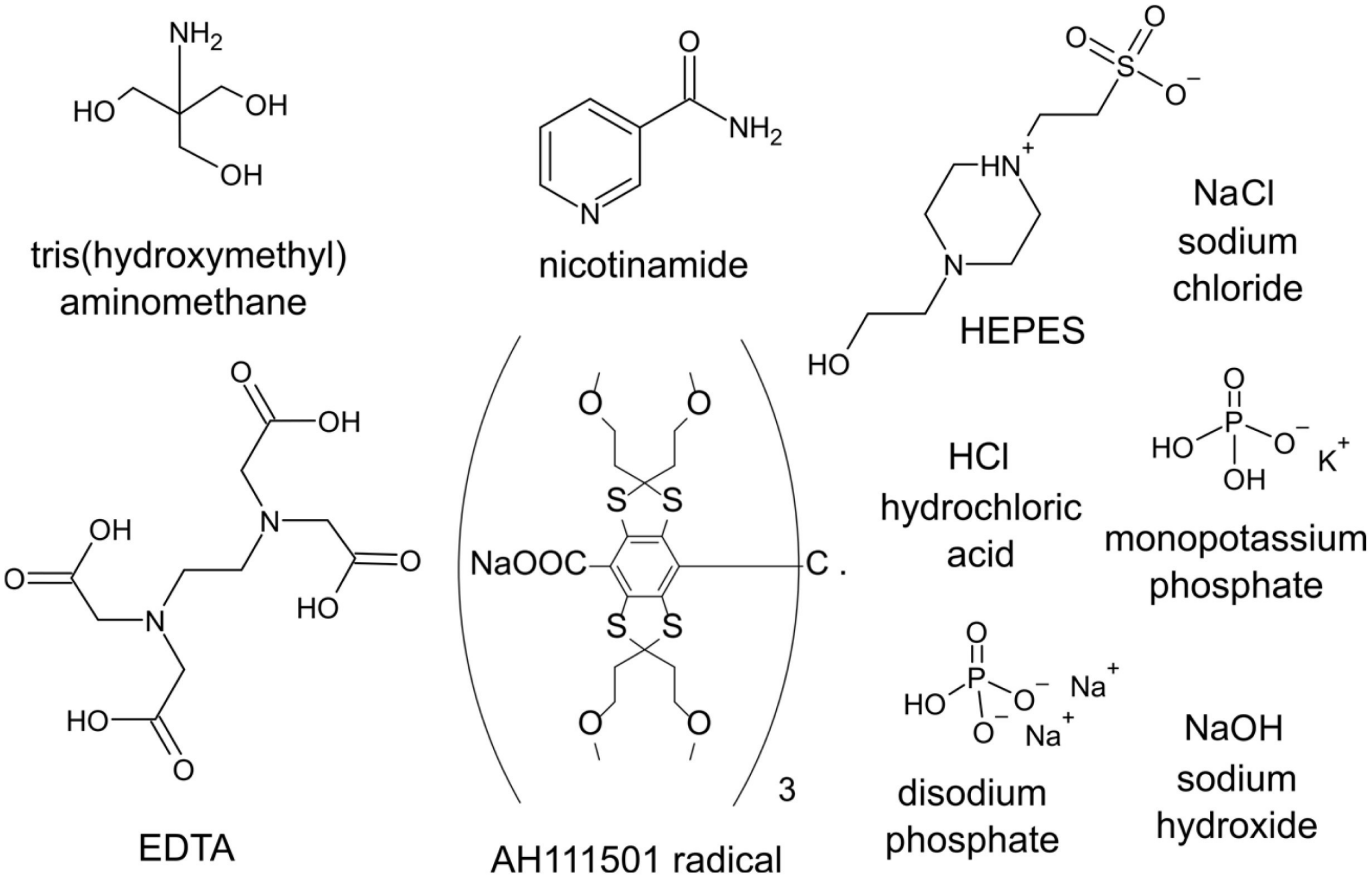
Structures of compounds added to [1‐^13^C]pyruvate aqueous solution. Monopotassium and disodium phosphate were part of the phosphate buffer solution (PBS). The solvent was a mixture of H_2_O (0%–90%) and D_2_O (10%–100%).

Here, we analyze and present the impact of chemicals, magnetic field, and preparation procedures on the ^13^C *T*_1_ of thermal and hyperpolarized [1‐^13^C]pyruvate. We used the MFC system coupled with a 9.4 T high‐resolution NMR [33] to gain information on the impact on relaxation associated with the addition (or removal) of chemicals (Figure 1). We examined the *T*_1_ relaxation of 35 different [1‐^13^C]pyruvate sample compositions each at 20–‍39 fields between 7.8 µT and 9.4 T (nuclear magnetic relaxation dispersion, NMRD) and estimated losses of polarization during manual transfer of the hyperpolarized pyruvate to the NMR spectrometer. We increased *T*_1_ up to 627% at 7.8 µT–8 mT and 375% at 0.1–1 T compared to pure [1‐^13^C]pyruvate in pure H_2_O [19].

Overall, this work systematically examines various additives and constituents of the pyruvate sample, building on our first assessment [19] and aiming to provide more comprehensive insights into sample optimization strategies and the underlying molecular interactions.

## 2 Results and Discussion

### 2.1 Overview

To determine the optimal conditions for the most commonly used hyperpolarized probe, [1‐^13^C]pyruvate [5], we examined its *T*_1_ as a function of *B*_0_ = 7.8 µT–9.4 T (NMRD), in H_2_O and D_2_O. We added combinations of trityl radical (AH111501), NaCl, EDTA, nicotinamide (NAM), phosphate‐buffered saline (PBS), tris(hydroxymethyl)aminomethane (Tris) buffer, or 4‐(2‐‍hydroxyethyl)‐1‐piperazineethanesulfonic acid (HEPES) buffer (Figure ‍1). All samples were at neutral pH and either degassed or at ambient gas content (samples contained at least 10% D_2_O for locking and shimming; see Table S1).

**FIGURE 2:**
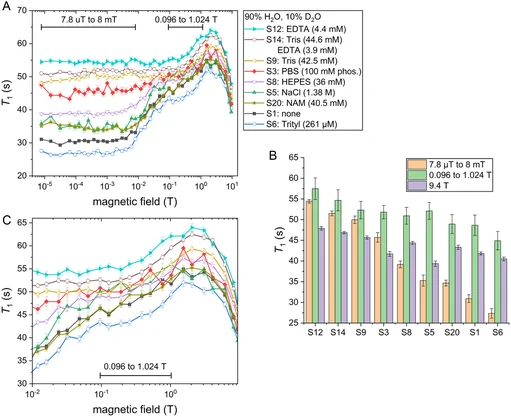
NMRD of [1‐^13^C]pyruvate at fields from 7.8 µT to 9.4 T with several additives in H_2_O. (A–C) *T*_1_ of [1‐^13^C]pyruvate as a function of magnetic field *B*_0_ for different additive combinations. Low, intermediate, and high field regimes were defined (B). The additives were found to be most effective in the low field range and least effective in the high field. H_2_O containing 261 µM AH111501 trityl radical (S6) resulted in the shortest *T*_1_^IF^ = (27.4 ± 1.2) s, while 4.4 mM EDTA (S12) provided the longest *T*_1_^IF^ = (54.4 ± 0.4) s. The longest relaxation times were observed at ~1 T (≥50 s), the shortest at Earth’s magnetic field (*T*_1_ ~ 30 s). Plotted error bars in panel (A) and (C) represent the SD of the exponential fit.

**FIGURE 3:**
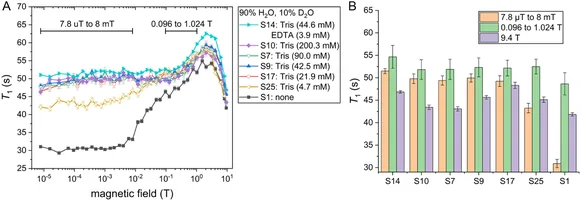
NMRD of [1‐^13^C]pyruvate at fields from 7.8 µT to 9.4 T in H_2_O for different concentrations of Tris. (A) T_1_ of [1‐^13^C]pyruvate as a function of magnetic field *B*_0_ for different additive combinations. Low, intermediate, and high field regimes were defined (B). *T*_1_^LF^ was increasing upon increase of [Tris] up to ~40 mM, while *T*_1_^HF^ was first increasing, peaking at 21.9 mM, and then falling at further [Tris] increase: 41.8 ± 0.4 s (0 mM), 48.3 ± 0.7 s (22 mM), 43.4 ± 0.5 s (200 mM). In all cases, the *T*_1_ across all fields was longer than for pure H_2_O (gray curve). The chemical shift of 1‐^13^C changes by −0.32 ppm per mol/L of Tris. Plotted error bars in panel (A) represent the SD of the exponential fit.

**FIGURE 4:**
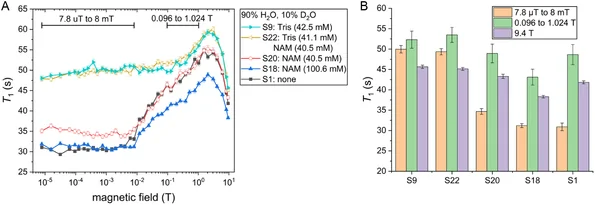
NMRD of [1‐^13^C]pyruvate in H_2_O with varying [Tris] and [NAM]. (A) T_1_ of [1‐^13^C]pyruvate as a function of magnetic field *B*_0_ for different additive combinations. Low, intermediate, and high field regimes were defined (B). Adding excessive amounts of NAM (100.6 mM) provided no observable benefit with 31.2 ± 0.5 s at LF (S18 vs. S1) and even a decrease at IF and HF. Combining Tris and NAM (S22) increased the *T*_1_ across the entire field range, and beyond that of the Tris‐only S9 at IF (53.5 vs. 52.3 s). Plotted error bars in panel (A) represent the SD of the exponential fit.

**FIGURE 5:**
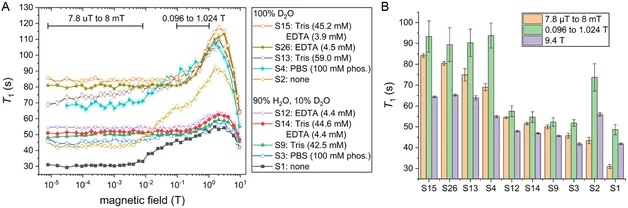
NMRD of [1‐^13^C]pyruvate with different additives in H_2_O and D_2_O. (A) T_1_ of [1‐^13^C]pyruvate as a function of magnetic field *B*_0_ for different additive combinations. Low, intermediate, and high field regimes were defined (B). A clear benefit of solvent deuteration was visible across all field ranges: without additives *T*_1_^LF^ rose from (30.9 ± 0.9) s in H_2_O (S1) to (43.4 ± 1.5) s in D_2_O (S2) and with Tris and EDTA from (51.5 ± 0.6) s (S14) to (84.2 ± 0.9) s (S15). Maximum *T*_1_ of (117.5 ± 1.5) s at 2 T was reached for the combination of Tris and EDTA in D_2_O (S15). Plotted error bars in panel (A) represent the SD of the exponential fit.

**FIGURE 6:**
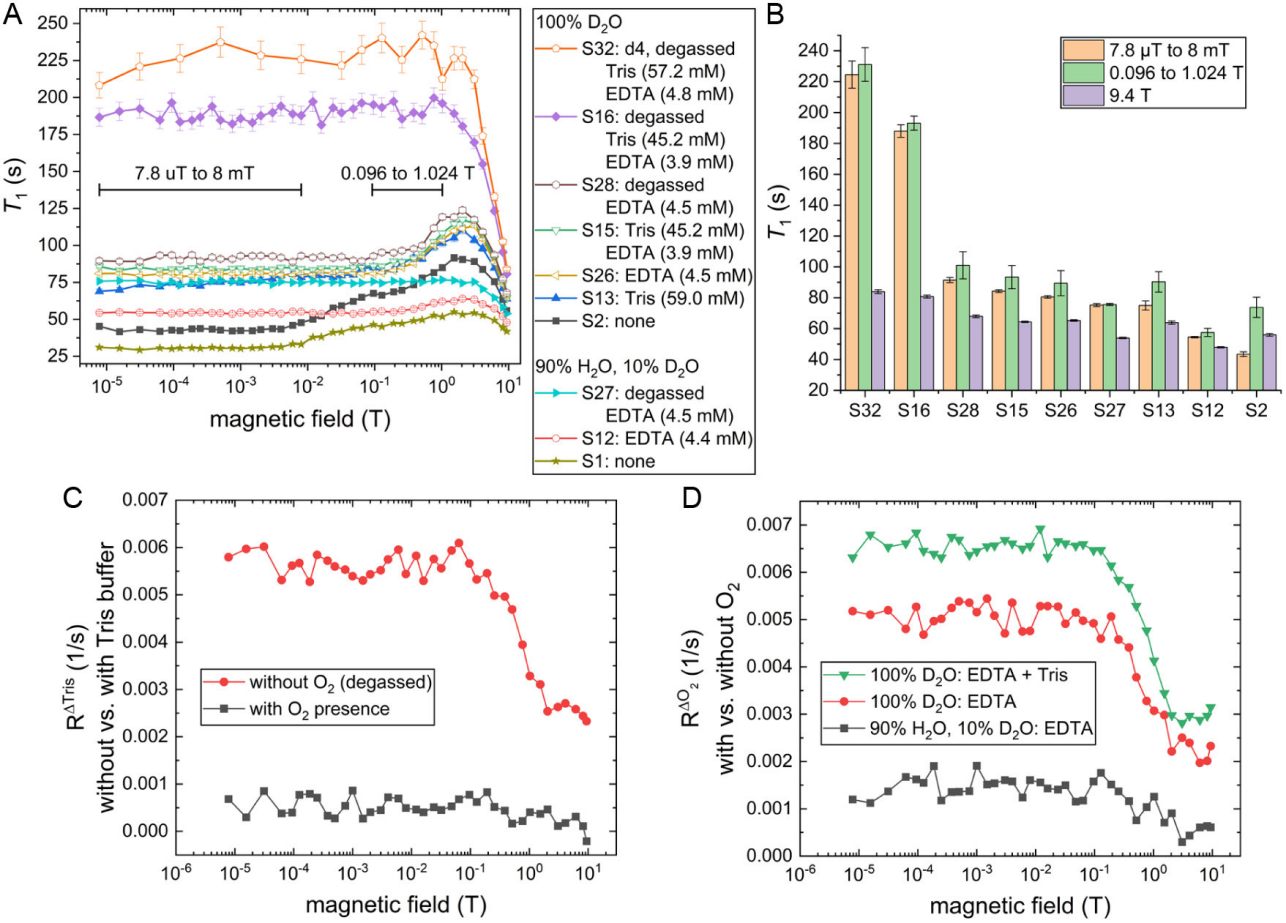
NMRD of [1‐^13^C]pyruvate with and without degassing and difference in relaxation rates depending on oxygen presence. (A) T_1_ of [1‐^13^C]pyruvate as a function of magnetic field *B*_0_ for different additive combinations. Low, intermediate, and high field regimes were defined (B). Significant difference in *T*_1_^LF^ can be observed between the different samples. Pure D_2_O (S2) experiences the shortest *T*_1_ of all D_2_O samples (43.4 ± 1.5 s), while the degassed D_2_O sample containing deuterated pyruvate, Tris, and EDTA (S32) provides the longest (224.5 ± 8.8 s). The S16 containing Tris buffer had a longer *T*_1_ (188.0 ± 4.1 s) compared to the degassed S28 without Tris (91.4 ± 1.7 s). In H_2_O, degassing improved *T*_1_^LF^ from 54.4 ± 0.4 s (S12) to 75.3 ± 1.0 (S27). *T*_1_^IF^ was highest for the S32 (231.1 ± 10.9 s), about constant for S28, S15, S26, S13, and S27, and again the shortest for S1 and S2. *T*_1_^HF^changed by the smallest absolute amount, although also about doubled between S1 (41.8 ± 0.4 s) and S32 (83.9 ± 1.2 s). The field between roughly 0.1 and 1 T gives the longest relaxation time across all samples and reached a maximum of 242.1 ± 9.4 s at 0.512 T for the S32. (C) Tris reduces the relaxation at low fields much more effectively for deoxygenated samples (red), adding Tris increased *T*_1_^LF^ from 91.4 ± 1.7 s (S28) to 188.0 ± 4.1 s (S16). In the presence of oxygen, *T*_1_^LF^ only improved from 80.5 ± 0.8 s (S26) to 84.2 ± 0.9 s (S15). (D) Removal of oxygen provides a bigger benefit when using 100% D_2_O as a solvent (red) instead of 90% H_2_O and 10% D_2_O (gray), and removal of oxygen provides the biggest benefit when Tris is present in 100% D_2_O (green). Plotted error bars in panel (A) represent the SD of the exponential fit.

**FIGURE 7:**
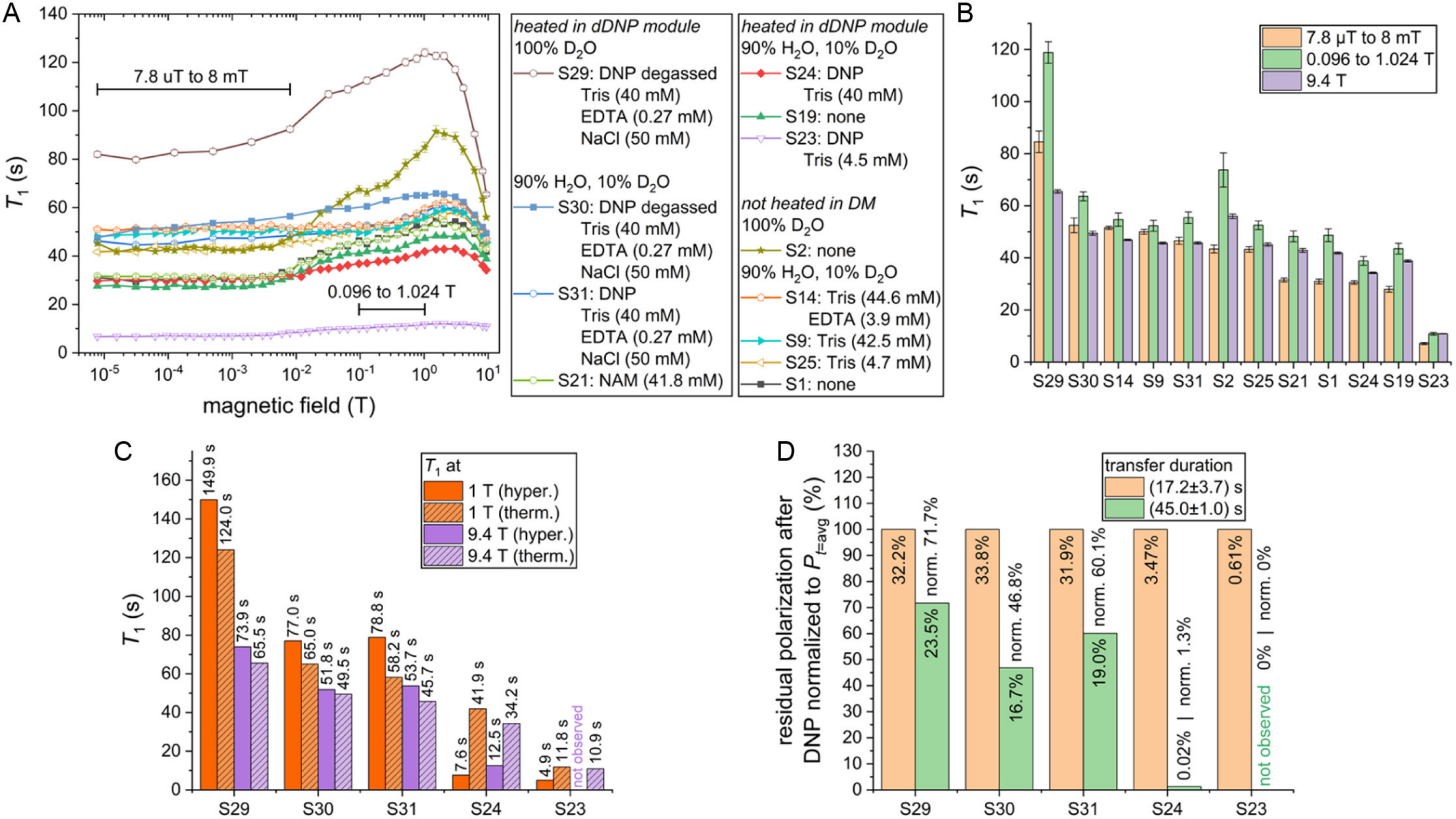
NMRD of [1‐^13^C]pyruvate at fields from 7.8 µT to 9.4 T in pure H_2_O and D_2_O or after hyperpolarization experiments following dissolution DNP. (A) T_1_ of [1‐^13^C]pyruvate as a function of magnetic field *B*_0_ for different additive combinations. Low, intermediate, and high field regimes were defined (B). The deuterated sample clearly shows a prolonged *T*_1_ compared to its 90% H_2_O counterpart (S29 vs. S30). Interestingly, in the DNP samples (S29, S30, S31, S24, S23), the decline below a 1 T field is gentler. The *T*_1_ of the DNP sample with only 4.5 mM Tris was the shortest (7.1 ± 0.4 s at low field), while the D_2_O degassed sample provided 84.6 ± 4.1 s. Respective samples without the dDNP process are plotted (S14, S9, S2, S25, S1) to provide a reference point. Addition of ~41 mM NAM (S21) restored *T*_1_ values to those of pure H_2_O (S1). (C) The hyperpolarized *T*_1_ acquired at 1 and 9.4 T systems were compared to the respective thermally acquired values on the same samples (S29, S30, S31, S24, S23). For the S29, S30, and S31, the thermal *T*_1_ was below the hyperpolarized *T*_1_, which was attributed to (i) a higher temperature following hyperpolarization; (ii) the helium used during heating and dissolution is believed to replace oxygen in the hyperpolarized sample, thereby prolonging *T*_1_. Therefore, in the case of S31, the difference is more pronounced because no oxygen removal by argon was conducted, and the helium was replaced by air over time. (D) The polarization of the hyperpolarized samples was quantified after (17.2 ± 3.7) s at 1 T and after (45.0 ± 1.0) s at 9.4 T. A clear difference in preserved polarization after 45 vs. 17 s can be observed depending on the sample composition. Variation in the 17 s polarization values of S29, S30, and S31 might also be due to slight variation in solid‐state polarization. Not surprisingly, the preserved polarization follows the hyperpolarized *T*_1_ rather than the thermally acquired one, leading to S31 preserving more polarization compared to S30. The *T*_1_ of S23 was too short to observe any signal after 45 s. Polarization was normalized to the average transfer duration shown in the legend in (D). Average pH was 7.5 ± 0.1. Plotted error bars in panel (A) represent the SD of the exponential fit.

**FIGURE 8:**
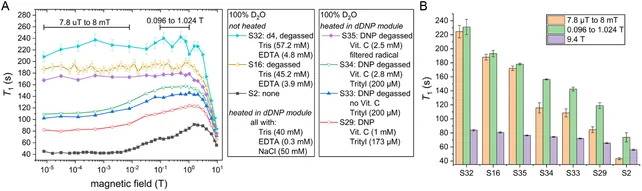
NMRD of [1‐^13^C]pyruvate dDNP samples and the impact of degassing, vitamin C, and radical filtration. (A) T_1_ of [1‐^13^C]pyruvate as a function of magnetic field *B*_0_ for different additive combinations. Low, intermediate, and high field regimes were defined (B). Significant difference in *T*_1_^LF^ can be observed between the DNP samples depending on oxygen removal, addition of vitamin C, and radical filtration. Successive treatment of a post‐dDNP sample raised *T*_1_^LF^ from (84.6 ± 4.1) s with degassing of the dissolution medium alone (S29) to (172.2 ± 3.1) s after argon bubbling, vitamin C and radical filtration (S35)—close to the (188.0 ± 4.1) s of the comparable S16, which never contained radical nor passed through the dissolution module, and short of the (224.5 ± 8.8) s reached with perdeuterated pyruvate (S32). Plotted error bars in panel (A) represent the SD of the exponential fit.

For each sample, relaxation decay kinetics were measured at various fields, and a biexponential decay function $A_{1}e^{-t/\tau_{1}}\;+\;A_{2}e^{-t/\tau_{2}}\;+\;y_{0}$ was fitted to the integral of the pyruvate signal as a function of time *t* at each sampled field. The constant with the largest amplitude *A*_1_, *τ*_1_ was associated with [1‐^13^C]pyruvate *T*_1_ and is used for the NMRDs (Figures 2–8). The second time constant *τ*_2_ was kept constant across all fields for each sample individually to compensate for the observed nonmonoexponentially decay; note that the contribution of *τ*_2_ to the signal was relatively low (*A*_2_ ≪ *A*_1_) or *A*_2_ = 0 for some samples. y_0_ was associated with thermal polarization in the given field. Fit parameters and experimental kinetics can be found in the supporting information spreadsheet file.

To estimate the “retained” polarization at the time of injection (*P*_ATOI_), we considered *T*_1_(*B*) and the magnetic field profile during sample transfer (Figure S1). Finally, we measured the retained hyperpolarization level experimentally using an optimized pyruvate sample and compared it with the previously used standard. The sample was hyperpolarized using dissolution dynamic nuclear polarization (dDNP) as described previously [36].

All investigations showed a very similar pattern of *T*_1_ as a function of *B*_0_: a range of *T*_1_(*B*_0_) with zero or small, constant slope, an increase in *T*_1_ with larger, constant slope, a short plateau, and a decrease with the steepest slope. We refer to the ranges between 7.8 µT and 8 mT as a low field (*B*_0_ = *B*_LF_), the field range between 0.1 and 1 T as an intermediate field (*B*_0_ = *B*_IF_), and 9.4 T as a high field (*B*_0_ = *B*_HF_). These ranges were chosen to represent the field without a transfer magnet (*B*_LF_), inside a transfer magnet (*B*_IF_), and in a high‐resolution spectrometer (*B*_HF_). The *T*_1_ within this range was relatively homogeneous, and the average *T*_1_ values, with their standard deviation (except for HF, SD of the fit), were reported for each range: *T*_1_^LF^, *T*_1_^IF^, or *T*_1_^HF^.

To shorten scan time in nonhyperpolarized experiments, ^1^H polarization was transferred to ^13^C using the INEPT (insensitive nuclei enhanced by polarization transfer) sequence [33, 37], as discussed previously [33]. This was applied to all samples except fully deuterated pyruvate. All 35 samples are referenced throughout the text using their unique IDs (sample compositions are in Table S1): S1–S35 across all figures and the supporting information Excel sheet.

### 2.2 NMRD of [1‐^13^C]Pyruvate in H_2_O: Screening of Additives for Impacting *T*_1_

During the first screening of additives (Figure 2), we found that the H_2_O sample with 261 µM of trityl showed the fastest *T*_1_^LF^ of (27.4 ± 1.2) s. Although trityl radical accelerates relaxation, as previously demonstrated [38], the relaxation times were only slightly shorter than pure H_2_O with *T*_1_^LF^ of (30.9 ± 0.9 ) s. Adding PBS dramatically increased *T*_1_^LF^ to (45.7 ± 1.1) s (50% gain in *T*_1_^LF^ compared to pure H_2_O). Across all experiments, we found that pure H_2_O with either EDTA, Tris, PBS, or a combination yielded the longest *T*_1_^LF^ = 45–55 s (Figure 2)—almost twice as long as without additives (pure H_2_O). HEPES buffer did not achieve the same *T*_1_^LF^ as other buffers (PBS or Tris), although it was still prolonged compared to pure H_2_O. Interestingly, the addition of NaCl or NAM changed *T*_1_^LF^, yielding about 4 s longer *T*_1_^LF^ than in pure H_2_O.

Across the additives screened in H_2_O, *T*_1_^LF^ varied by a factor of two while *T*_1_^HF^ varied by less than 25%, i.e., the additives act most strongly where relaxation is fastest. *T*_1_^LF^ correlates with *T*_1_^IF^ and *T*_1_^HF^ (Pearson *r* = 0.90, 0.82 and 0.71 for LF–IF, LF–HF and IF–HF, Figure 2), so a low‐field measurement has some predictive power, but the full NMRD remains necessary to resolve the individual contributions.

### 2.3 H_2_O: [Tris] Impact on *T*_1_

Motivated by the finding that Tris had a large effect on *T*_1_, we varied its concentration [Tris] = 4.7–200.3 mM (in a typical dDNP experiment, [Tris] ~ 40 mM). We found that *T*_1_ was increased from *T*_1_^LF^([Tris] = 0 mM] = 30 s to *T*_1_^LF^(4.7 mM) = 43 s and *T*_1_^HF^(0 ‍mM) = 43 s to *T*_1_^HF^(4.7 mM) = 45 s. For higher concentrations, *T*_1_ increased further *T*_1_^LF^(21.9 mM) = 49 s and *T*_1_^HF^(21.9 ‍mM) = 48 s (Figure 3), while 200 mM led to a reduction of *T*_1_^HF^(200.3 mM) = 43 s. *T*_1_ was always longer with Tris than without (for the concentrations investigated). Adding 3.9 mM EDTA to 44.6 mM Tris further increased *T*_1_ across all fields compared with the Tris‐only samples, albeit by only 2–10 s.

### 2.4 H_2_O: [NAM] Impact on *T*_1_

Following the observation of a positive effect of NAM addition on pyruvate (Figure 2) and ^15^N probes shown elsewhere [15], we assessed its impact on [1‐^13^C]pyruvate and when combining it with Tris (Figure 4). Adding 40.5 mM NAM (to pure H_2_O) increased *T*_1_ at low fields by about 4 s and had no effect at high field (red and black, Figure 4). Further increase to [NAM] = 100 mM decreased *T*_1_^IF^ and *T*_1_^HF^ below those of pure H_2_O, and *T*_1_^LF^ to the level of the H_2_O sample. This decrease aligns with the observations made with Tris explained within the CIDER mechanism (see previous section). The achieved low‐ and high‐field values remain much lower than those obtained with ~40 mM Tris, and no additional benefit of adding NAM to Tris was apparent.

### 2.5 Impact of D_2_O and H_2_O as Solvent

Replacing H_2_O as a solvent with D_2_O increased *T*_1_^LF^ ‍= ‍(30.9 ± 0.9) s by more than 40% to (43.4 ± 1.5) s (Figure ‍5, S2 vs. S1) and *T*_1_^HF^(H_2_O) = 41.8 s by more than 30% to *T*_1_^HF^(D_2_O) = 55.9 s. Adding PBS, Tris, EDTA, and combinations thereof almost doubled *T*_1_ again at low and intermediate field (e.g., *T*_1_^LF^(H_2_O) = 31 s, *T*_1_^LF^(D_2_O) = 45 s, *T*_1_^LF^(D_2_O, EDTA) = 80 s (Figure 5)). The addition of Tris and EDTA, which both may act as CIDER additives and chelate impurities, increased *T*_1_^IF^(D_2_O, Tris) = (74.9 ± 3) s and *T*_1_^LF^(D_2_O, EDTA) = (80.5 ± 0.8) s compared to (50.0 ± 0.9) s and (54.4 ± 0.4) s, respectively, in H_2_O (Figure 5, S13 and S26 vs. S9 and S12). Note that H_2_O and D_2_O may contain different amounts of (unknown) paramagnetic impurities.

### 2.6 D_2_O and H_2_O: Effect of O_2_

To assess the impact of O_2_, we removed the air by first applying 3 min of argon bubbling and then three freeze‐pump‐thaw cycles to EDTA in D_2_O (S28) and in H_2_O (S27, Figure 6). Details about the degassing are provided in SI. Degassing boosted *T*_1_^LF^ to 91 s in D_2_O and 75 s in H_2_O from 81 and 54 s, respectively. Degassing the sample containing Tris and EDTA in D_2_O (S16) using three freeze‐pump‐thaw cycles more than doubled *T*_1_^LF^ from 84 to 188 s, demonstrating a strong impact of oxygen‐induced paramagnetic relaxation on [1‐^13^C]pyruvate.

The difference in relaxation rates between the native aqueous solution and the degassed one allowed us to estimate the oxygen impact: $R_{1}^{\text{Δ}O_{2}}=R_{1}‐R_{1}^{\mathrm{degassed}}$ (Figure 6D). $R_{1}^{\text{Δ}O_{2}}$ can be used to estimate the impact of sample degassing when such a process is not performed, for example, the impact of degassing the dDNP dissolution medium. $R_{1}^{\text{Δ}O_{2}}$ varied up to fivefold with solvent and additive: ~0.0015 s^−1^ in H_2_O, ~0.0050 s^−1^ in D_2_O, and ~0.0065 s^−1^ in D_2_O with Tris. Correspondingly, the deceleration provided by Tris rose from below 0.001 s^−1^ in oxygenated D_2_O to ~0.006 s^−1^ once oxygen was removed.

### 2.7 H_2_O and D_2_O: Impact From dDNP‐Induced Impurities

To investigate potential impurities induced by the DNP process, we used H_2_O or D_2_O after heating it in the dDNP dissolution chamber. This part included the normal heating of the solvents inside the DNP (incl. a stainless‐steel heater), but without dissolution of a hyperpolarized sample—pyruvate was added afterwards like for all previous samples (S21 and S19, Figure 7). In parallel, we performed full DNP experiments with the given sample compositions in the dissolution medium to compare *T*_1_ of thermally and hyperpolarized pyruvate (S29, S30, S31, S24, S23 indicated with “DNP”).

Interestingly, after heating the same H_2_O in the dissolution module of the dDNP, the *T*_1_^LF^ decreased from (30.9 ± 0.9) s to (27.9 ± 1.1) s (S1 vs. S19). The addition of ~40 mM of NAM (S21) restored the *T*_1_ values to levels above those of the pure H_2_O sample. We observed that hyperpolarized *T*_1_ was longer than the thermally acquired *T*_1_ of the same sample (for example, see S24, which shows a multiple‐fold difference; Figure 7C).

During the DNP process, the dissolution medium was pressurized with approximately 5 bar of helium before heating (SpinAligner, Polarize), thereby reducing the fraction of dissolved air relative to the sample at ambient pressure. The noble gases would then diffuse out of the sample over time after dissolution, leaving the resulting thermal sample exposed to air.

We degassed the dissolution medium, filled the NMR tube with argon gas, and sealed the sample right after dissolution, reducing the disparity in *T*_1_ in S30 between hyperpolarized and thermally polarized samples to below 20% at 1 T and below 5% at 9.4 T, suggesting that indeed the reduced oxygen fraction after dissolution leads to longer observed hyperpolarized *T*_1_. The remaining gain in *T*_1_ could be attributed to a slight increase in the sample temperature after dissolution.

To demonstrate the benefits of prolonged *T*_1_ from optimized sample compositions, hyperpolarized samples were measured after (17.2 ± 3.7) s at 1 T and after (45.0 ± 1.0) s at 9.4 T. While each HP sample composition was measured once, the transfer time is the average of all 5 experiments. In particular, the retained polarization after long transfer reflected the extended hyperpolarized 1 T *T*_1_, with S29 in D_2_O retaining ~72% and S30 in H_2_O retaining ~47% of the polarization measured with the short transfer time; similar polarization values were observed after short transfer.

### 2.8 dDNP of [1‐^13^C]Pyruvate: Tris Case Study

We performed dDNP experiments with and without Tris buffer to observe its impact. However, 4.5 mM rather than 0 mM was chosen as the lowest [Tris]: it was the minimum concentration at which successful buffering of the hyperpolarized solution was achieved (about 22:1 [pyruvate]:[Tris]); otherwise, the pH would fluctuate between pH values 4 and 10 due to slight sample composition variation.

In these experiments, the 4.5 mM Tris experiment resulted in 0.61% polarization after a 15.2 s transfer, with a *T*_1_ of 4.9 s at 1 T and a pH of 7.42 (Figure 7, S23). No residual polarization was detected at 9.4 T after a 43.9 s transfer. At 40 mM concentration, however, the polarization was 3.5% after 12 s transfer with a 7.6 s *T*_1_ at 1 T and pH 7.33; a 12.5 s *T*_1_ was observed at 9.4 T after a 45.2 s transfer (Figure 7, S24). In both cases, no EDTA was added to avoid any influence on the results; however, this led to a reduction in polarization of about 90% after 17 s compared to a typical dDNP experiment (Figure 7, S31). These results showcase how different buffer concentrations affect the *T*_1_ and thus residual polarization in a real‐world setting and how the sample conditions following dDNP are quite different to those in pure H_2_O, where 1 T *T*_1_ at 42.5 mM Tris concentration at pH 7.6 was (56.0 ± 0.7) s, so about 7 times longer than after dDNP (Figure 7, S9 vs. S24). This demonstrates the detrimental effect of the Trityl radical and other paramagnetic impurities on the hyperpolarized probe if they are not removed or isolated from it.

### 2.9 NMRD of [1‐^13^C]Pyruvate DNP Samples: Impact of Degassing, Vitamin C, and Radical Filtration

To understand the impact of degassing, addition of vitamin C, and radical filtration on previously hyperpolarized dDNP samples, formerly hyperpolarized samples were stored and modified accordingly before the NMRD measurement (Figure 8). Degassing was performed using argon bubbling for S35, S34, S33, and S29 over 5 min after dDNP dissolution or using argon bubbling (S32) and/or pump‐thaw‐cycles (S32 and S16).

The degassing of the DNP sample increased *T*_1_^LF^ from (84.6 ± 4.1) ‍s to (115.6 ± 7.3) s‍‍ (S29 vs. S34), addition of vitamin C from (108.5 ± 5.8) s to (115.6 ± 7.3) s (S33 vs. S34), and further filtration of radical from (115.6 ± 7.3) s to (172.2 ± 3.1) s (S34 vs. S35). In contrast, S16—which never contained radical and never passed through the heated dissolution module—reached (188.0 ± 4.1) s, and deuterating the pyruvate extended it to (224.5 ± 8.8) s (S32). Even combined, the post‐hoc treatments did not quite close that gap (maximum of 172.2  ±  3.1 s for S35), indicating that dDNP leaves a residual relaxation contribution which they only partly remove.

### 2.10 Proposed Molecular Interactions

Four interactions are consistent with the observations above; we distinguish those that follow from the data from those that remain speculative.

#### 2.10.1 Hydrogen Bonding Between Buffer and Probe

Tris, and to a lesser extent NAM, extended *T*_1_^LF^ up to an optimum concentration and shortened it beyond (Figures 3, 4). Within the CIDER framework [15], hydrogen bonding increases the minimal distance at which paramagnetic impurities approach pyruvate, extending *T*_1_^LF^, while the increased effective molecular size lengthens the rotational correlation time and thereby shortens *T*_1_^HF^. The opposing field dependence of these two effects accounts for the observed concentration optimum.

#### 2.10.2 Dipolar Coupling to Solvent Protons

Replacing H_2_O with D_2_O extended *T*_1_ at every field and for every additive tested (Figure 5), indicating a substantial intermolecular dipole–dipole contribution from the solvent.

#### 2.10.3 *A Tris‐O*_2_ *‐Pyruvate Interaction*

The oxygen and Tris contributions were not additive: Tris decelerated relaxation far more effectively once oxygen had been removed (Figure 6C), and $R_{1}^{\text{Δ}O_{2}}$ was Tris‐dependent and largest in the presence of Tris (Figure 6D). The big difference in $R_{1}^{\text{Δ}O_{2}}$ in H_2_O and D_2_O could not be explained by a marginal difference in oxygen diffusion and solubility [39, 40] and deserves further investigation.

#### 2.10.4 Trace Metal Cations

Water heated to 200 °C in our steel dissolution module shortened *T*_1_ across the full field range (S19), and NAM restored it (S21), consistent with cations released during heating and subsequent protection of the probe.

### 2.11 Machine Learning: Optimized Sample Composition

To predict *T*_1_ relaxation times across low, medium, and high field strengths, we employed a machine learning approach using Gradient Boosting Regressor (GBR) ensembles. This algorithm was selected to capture nonlinear relationships between chemical additives and relaxation rates. The models were trained on experimental data comprising 11 chemical features, including explicitly defined interaction terms (e.g., D_2_O * O_2_ and D_2_O * Tris) to account for synergistic effects. We utilized an ensemble of 300 decision trees with a maximum depth of 3 to balance model complexity and generalization, validated via 5‐fold cross‐validation. Following model training, a global differential evolution optimization was performed to identify the specific concentrations of Tris, EDTA, NAM, NaCl, and vitamin C that maximize *T*_1_ at each field strength. A detailed description of the data preprocessing, model hyperparameters, and SHAP explainability analysis is provided in the Supporting Information.

Three optimized formulations were obtained. For low field, the composition was 80.3 mM Tris, 4.7 mM EDTA, 66.3 mM NAM, 363.5 mM NaCl, and 2.4 mM vitamin C, with predicted *T*_1_^LF^ = 231.6 s. For the medium field, it is 62.1 mM Tris, 4.8 mM EDTA, 42.6 mM NAM, 1429.2 mM NaCl, and 2.1 mM vitamin C, with predicted *T*_1_^IF^ = 237.2 s. For high field, it is 57.5 mM Tris, 3.0 mM EDTA, 47.3 mM NAM, 443.8 mM NaCl, and 4.9 mM vitamin C, with predicted *T*_1_^HF^ = 86.9 s. These values are reasonably close to the S32 from Figure 8 with a *T*_1_ of 224.5, 231.1, and 83.9 s at LF, IF, and HF, respectively. This small difference suggests that the achieved values in this work are close to the expected maximum for the current sample composition.

### 2.12 Limitations

The optimal sample composition is probe‐specific: CIDER‐type deceleration relies on hydrogen bonding between additive and probe, so while translation to [2‐^13^C]pyruvate should be straightforward, the additive and its concentration must be re‐determined for each probe—more strongly charged substrates such as fumarate or succinate may require different ones. We have not identified the mechanism underlying the buffer effect, so extrapolation beyond the compositions measured here is not warranted. Results are also platform‐ and site‐dependent: heating water in our stainless‐steel dissolution module alone shortened *T*_1_^LF^ (S19 vs. ‍S1), and the estimated losses follow our own transfer field profile. Finally, the longest‐lived samples (S16, S32) contained no radical and were flame‐sealed after freeze‐pump‐thaw degassing, and the numerical optimization was not constrained to biocompatible concentrations (1.4 M NaCl at intermediate field); these are an upper bound on achievable *T*_1_ rather than an injectable formulation, for which the compatible route is deuteration and degassing of the dissolution medium with radical filtration (S35).

## 3 Conclusion and Outlook

We observed that the low‐field relaxation of pyruvate was strongly affected by buffers. Previously, this effect of increased *T*_1_ time was observed for ^15^N‐containing molecules and was attributed to shielding from paramagnetic impurities and slowing of chemical exchange: Tris buffer has structural elements similar to the CIDER additive glycerol, which was examined previously [15]. Here, 90 mM Tris buffer increased *T*_1_^LF^ of pyruvate from 30.9 s in pure water to 49.4 s (Figure 3, S7 vs. S1). Other buffers, such as PBS and HEPES, also prolonged *T*_1_^LF^. While Tris buffer is used regularly in dissolution media for dDNP, HEPES, which contains a sulfur group, may decompose into toxic compounds when heated. These buffers have been used previously to adjust the pH to the physiological range—our work, however, demonstrates that they also provide additional benefits by prolonging *T*_1_. Further studies may aim to fine‐tune the optimal buffer based on pyruvate concentration to maximize *T*_1_.

The availability of the NMRD helps to understand and to design the optimal hyperpolarization experiment. The results suggest conducting the critical steps of purification or sample transfer in the relatively high fields of ~1 T, ideally using the composition with the longest relaxation time. Pyruvate is not very sensitive to trityl (at concentrations below 1 mM) after dDNP dissolution in an aqueous, Tris‐buffered solution with EDTA, a typical dDNP sample composition. This explains the high polarization values reported for dDNP even when the radical is not filtered from the solution ― for maximizing polarization, performing no radical removal can actually be beneficial [19]. In all examined cases, the field between 0.1 and 2 T yielded the highest ^13^C *T*_1_ for [1‐^13^C]pyruvate; hence, it should be used when possible, for manipulations of hyperpolarized pyruvate. Estimations of polarization losses for all samples are given in, Figure S2.

In practical terms, the optimized dDNP formulation S35 is expected to retain 84% of its polarization after a 30 s transfer at Earth’s field and 79% after 40 s, compared with 53% and 42% for a typical unoptimized preparation S31 in H_2_O‐based dissolution medium with Tris and EDTA and no degassing—a 1.6‐ to 1.9‐fold gain in delivered polarization, corresponding to a threefold reduction in transfer losses. These estimates are derived from the thermal *T*_1_ values; the hyperpolarized experiments support the trend directly, where S29 retained 72% and S31 60% of the polarization measured after the short transfer (Figure 7D). Using a degassed D_2_O dissolution medium, without radical filtration, already accounts for a 1.3‐ to 1.5‐fold gain (S31 vs. S29) and requires no change to the workflow beyond sample preparation.

This data concerns the relaxation up to in vivo injection. Still, considerable time passes afterward. The NMRD data suggest that relaxation is drastically decreasing below the range of human MRI devices (<1.5 T). Here, clinical scanners at 1.5–3 T are expected to have the longest relaxation in vivo, with most major vendors focusing their ^13^C options on 3 T. Experiments in whole blood or in vivo will provide further insights.

Some other molecules experience rapid polarization losses at low fields and some specific pH [14, 15, 16, 17, 18]. Investigations of the corresponding NMRD could shed light on the relaxation mechanism, how this rapid relaxation can be circumvented, and under what conditions the relaxation time reaches a maximum. Of the factors examined here, solvent deuteration, removal of paramagnetic impurities, and storage at 0.1–2 T act on the probe itself and could be used not only in dDNP but also in parahydrogen‐based hyperpolarization experiments [41, 42, 43]; buffer additives, however, should be used with caution, as they may compete for coordination at the metal center [44, 45, 46]. Dissolved oxygen plays a different role there: parahydrogen bubbling purges it during polarization, so the risk lies in re‐equilibration with air during subsequent handling.

Considering the progress in PHIP and SABRE, low fields even below those ~10 µT studied here are of interest; at such fields, spin order transfer from parahydrogen to the target happens spontaneously; however, considering the intricate behavior of the strongly coupled spin systems, their relaxation properties should be studied separately in relevant solvents and in the presence of catalyst [47].

## Author Contributions

**Josh P. Peters**: writing, final versions. **Jan‐Bernd Hövener**: conceptualization, MFC investigation, analysis, writing – original draft, writing, final versions, supervision, funding acquisition. **Andrey N. Pravdivtsev**: conceptualization, MFC investigation, analysis, writing – original draft, writing, final versions, supervision, funding acquisition. All authors contributed to discussions and interpretation of the results and have approved the final version of the manuscript.

## Funding

This study was supported by the German Federal Ministry of Education and Research (03WIR6208A hyperquant), the Heisenberg DFG grant (565789098), the Deutsche Forschungsgemeinschaft (562203308, 555951950, 527469039, HO‐4602/2‐2, HO‐4602/3, HO‐4604/6‐1, HO‐4604/8‐1, Inst 257/747‐1, EXC2167/2, FOR5042, TRR287), the European Regional Development Fund (122‐09‐053), and the Studienstiftung des Deutscher Volkes.

## Conflicts of Interest

The authors declare no conflicts of interest.

## Supporting information

Additional materials, including methods of sample preparation, acquisition, and machine learning, as well as estimations of polarization losses during transfer, are available online (.pdf). All NMRD kinetics and fit parameters are available in the supplementary spreadsheets all_NMRD_kinetics.xlsx and all_NMRD_fit.xlsx. These, together with the complete list of sample compositions and the corresponding *T*_1_ values and standard deviations (all_samples.xlsx), are deposited on Zenodo (https://doi.org/10.5281/zenodo.19455701).

## Data Availability

The data that support the findings of this study are openly available in Zenodo at https://doi.org/10.5281/zenodo.19455701, reference number 19455701.
