# Supplementary material for: Exploring Limits of [1‐13C]Pyruvate Nuclear Spin Relaxation: Impact of Field, Deuteration, Degassing, and Additives
Source: Small Sci. 2026 Aug 30;6(9):e70391. doi: 10.1002/smsc.70391 (PMC13533092; doi:10.1002/smsc.70391)
Supplement: Supplementary file 1 [file SMSC-6-e70391-s001.pdf]

Supporting Materials for

Exploring limits of [1-^{13}C]Pyruvate Nuclear Spin Relaxation: Impact of Field, Deuteration, Degassing, and Additives

Josh P. Peters[1]*, Jan-Bernd Hövener[1], Andrey N. Pravdivtsev[1]*

1. Section Biomedical Imaging (SBMI), Molecular Imaging North Competence Center (MOIN CC), Department of Radiology and Neuroradiology, University Hospital Schleswig-Holstein, Kiel University, Am Botanischen Garten 18, 24118 Kiel, Germany

* corresponding authors josh.peters@rad.uni-kiel.de, andrey.pravdivtsev@rad.uni-kiel.de

Content

1. Methods 2
   1.1. Sample compositions 2
   1.2. INEPT enhanced NMRD experiment 2
   1.3. Hyperpolarization and NMR signal acquisition 3
   1.4. Software 3
   1.5. Machine learning 3
2. [1-^{13}C]pyruvate polarization losses during transfer 5
   2.1. Magnetic field profile 5
   2.2. NMRD fitting 5
   2.3. Relaxation of hyperpolarization 6
   2.4. Retained polarization after transfer 6
3. References 9

1. Methods

1.1. Sample compositions

90.3±0.6 mM [1-^{13}C]pyruvate (molecular weight 89.05 g/mol, Sigma-Aldrich, 677175, CAS: 99124-30-8) for non-dDNP samples (98.4±1.6 mM for dDNP, n=8) with pH of 7.6±0.1 in 90% in H2O (Carl Roth, ID 3478.1) and 10% D_2O (Deutero, 00506) or 100% D_2O were used. The following chemicals were added: Trityl (Polarize, AH111501), NaCl (Sigma-Aldrich, S98881), Trizma (Tris) buffer (Sigma-Aldrich, T7943), ethylendiamintetracetic acid (EDTA, SERVA, 11280.02), HEPES (40 mM, Sigma-Aldrich, PHG0001), PBS (gibco, 70011-044), NAM (Sigma-Aldrich, 72340). NaOH (Carl Roth, 9356.1) and HCl (TCI Chemicals, H1202) were used to adjust the pH to the desired value.

Filtration of the radical from sample S35 was conducted by adding conc. HCl to the dDNP sample after dissolution, followed by filtration of the radical using a 0.2 µm filter (Chromafil Xtra, PET 20/25), and addition of NaOH to re-neutralize the sample. The solution before filtration is yellow-green, while after filtration, it was clear.

Degassing of the samples was conducted by three iterations of freezing in liquid N_2 followed by vacuuming and slow thawing for non-dDNP samples. The NMR tube was then flame-sealed under vacuum. The dDNP samples were degassed for 3 minutes using argon bubbling after dissolution, then closed with a standard NMR cap and parafilm.

***Tab. S1: sample compositions** of samples used for Figs. 2-8 in the main manuscript. The full list is also available as supplementary excel file (all_samples.xlsx).*

Sample	ID	pH	H2O (%)	D2O (%)	stock solution?	size (µL)	[pyruvate] (mM)	heated in DM?	degassed?	[trityl] (µM)	additive_1	[additive_1] (mM)	additive_2	[additive_2] (mM)	additive_3	[additive_3] (mM)	additive_4	[additive_4] (mM)	additive_5	[additive_5] (mM)
1	105	7.60	90	10	no	550	90.94	no	no	0										
2	106	7.55	0	100	no	550	90.75	no	no	0										
3	107	7.80	90	10	no	550	90.75	no	no	0	KCl	27	NaCl	1370	Na2HPO4	100	KH2PO4	18		
4	108	7.73	0	100	no	550	91.68	no	no	0	KCl	27	NaCl	1370	Na2HPO4	100	KH2PO4	18		
5	109	7.36	90	10	no	550	90.75	no	no	0	NaCl	1380								
6	116	7.55	90	10	no	550	90.19	no	no	261.3										
7	131	7.66	90	10	no	550	89.63	no	no	0	Tris	89.99								
8	132	7.68	90	10	no	550	90.37	no	no	0	HEPES	36								
9	135	7.68	90	10	no	550	89.81	no	no	0	Tris	42.51								
10	136	7.66	90	10	no	550	91.12	no	no	0	Tris	200.34								
11	137	7.62	90	10	no	550	89.63	no	no	0	EDTA	49.74								
12	138	7.62	90	10	no	550	90.75	no	no	0	EDTA	4.38								
13	139	7.55	0	100	no	550	89.25	no	no	0	Tris	58.95								
14	140	7.58	90	10	no	550	89.25	no	no	0	Tris	44.63	EDTA	4.38						
15	141	7.60	0	100	no	550	90.04	no	no	0	Tris	45.19	EDTA	3.88						
16	142	7.60	0	100	no	550	90.04	no	3x vaccum	0	Tris	45.19	EDTA	3.88						
17	144	7.70	90	10	no	550	90.75	no	no	0	Tris	21.92								
18	145	7.61	90	10	no	550	90.37	no	no	0	NAM	100.59								
19	146	7.71	90	10	DM stock #2	550	90.00	yes	no	0										
20	147	7.61	90	10	no	550	90.56	no	no	0	NAM	40.53								
21	148	7.60	90	10	DM stock #2	550	90.19	yes (w/o NAM)	no	0	NAM	41.76								
22	149	7.67	90	10	no	550	90.37	no	no	0	TrisB	41.05	NAM	40.53						
23	152	7.42	90	10	no	550	94.40	yes	no	166	TrisB	4.5								
24	153	7.33	90	10	no	550	100.10	yes	no	175	TrisB	40								
25	155	7.66	90	10	no	550	90.56	no	no	0	TrisB	4.66								
26	156	7.63	0	100	Pyr stock #280725_D2O	550	90.04	no	no	0	EDTA	4.5								
27	157	7.68	90	10	Pyr stock #280725_H2O	300	90.21	no	3 min argon + 3x vacuum	0	EDTA	4.51								
28	158	7.63	0	100	Pyr stock #280725_D2O	300	90.04	no	3 min argon + 3x vacuum	0	EDTA	4.5								
29	159	7.48	0	100	Pyruvate DM	550	98.60	yes	DM 5 min argon	173	Tris	40	NaCl	50	EDTA	0.27	NaOH	50	Vitamin C	1
30	160	7.70	90	10	Pyruvate DM	550	98.60	yes	DM 5 min argon	173	Tris	40	NaCl	50	EDTA	0.27	NaOH	50	Vitamin C	1
31	161	7.33	90	10	Pyruvate DM	550	98.60	yes	no	173	Tris	40	NaCl	50	EDTA	0.27	NaOH	50		
32	162	7.69	0	100	Pyr stock #140825_D2O	300	90.14	no	3 min argon + 3x vacuum	0	Tris	57.15	EDTA	4.76						
33	163	7.86	0	100	JAM422 & JAM423	550	98.90	yes	5 min argon after	200	Tris	40	NaCl	50	EDTA	0.27	NaOH	50		
34	164	7.80	0	100	JAM422 & JAM423	550	98.90	yes	5 min argon after	200	Tris	40	NaCl	50	EDTA	0.27	NaOH	50	Vitamin C	2.84
35	165	7.86	0	100	JAM422 & JAM423	550	98.90	yes	5 min argon after	200 (0 filtered)	Tris	40	NaCl	50	EDTA	0.27	NaOH	50	Vitamin C	2.46

1.2. INEPT enhanced NMRD experiment

The INEPT sequence[1] with refocusing was used without changes and without phase cycling:

^{13}C: ------τ-180_X-τ-90_X-τ-180_X-τ-90_Y-(shuttling)-90_X-FID

^{1}H: -90_X-τ-180_X-τ-90_Y-τ-180_X-τ------(shuttling)-decoupling

The optimum INEPT interpulse delay, τ, was calibrated for each sample experimentally. Subsequent MFC experiment followed the following sequence: (1) ^{1}H thermal relaxation at B_0 for D_{B0} = 19 s, (2) INEPT sequence to transfer ^{1}H polarization to longitudinal polarization of [1-^{13}C] of pyruvate, (3) shuttling to desired low-field, either inside the NMR bore or in the solenoid electromagnet (SE) within the magnetic shield and with appropriately set current, (4) waiting for ^{13}C relaxation at low-field for D_{LF}, (5) shuttling to B_0 followed by settling delay of 100 ms, (6) ^{13}C 90° excitation and signal acquisition with ^{1}H decoupling.

All extracted NMRD values are attached as supplementary excel file (all_NMRD_fit.xlsx and all_NMRD_kinetics.xlsx).

1.3. Hyperpolarization and NMR signal acquisition

NMR and MRI. ^{13}C MR signals were acquired using a 9.4 T wide-bore NMR (WB400, Avance NEO, Bruker) with a 5 mm BBFO probe. The NMR system temperature was set to 293 K, which is room temperature. Hyperpolarized samples were additionally measured at 1 T ^{13}C benchtop NMR (Spinsolve Carbon, Magritek).

dDNP sample. Samples for dDNP were prepared by mixing 14 M [1-^{13}C]pyruvate with 32 mM trityl radical. 19.5 µL of the mixture was used for hyperpolarization.

dDNP dissolution medium. The dissolution medium was prepared by mixing 1.51 g of Trizma pre-set crystals, 27 mg of EDTA, 0.756 g of NaCl, and 0.81 g of NaOH in 250 mL of water (90% H_2O and 10% D_2O or 100% D_2O). About 3.7 mL of dissolution medium was used. Degassing and addition of 1—2.5 mM vitamin C (Sigma-Aldrich, A0278) were performed directly before the experiment for the required samples. No transfer magnet was used.

1.4. Software

TopSpin 4.4.0 (Bruker), SpinsolveExpert 2.02.00 (Magritek), and magnetic-field-cycling hardware and software described in Peters et al. (2025, doi: 10.1021/acsmeasuresciau.5c00131) were used for data collection. Mestrenova 15.1.0-38027 (Mestrelab Research S.L.), and OriginPro 9.8.5.201 2021b (OriginLab Corporation) were used for data analysis.

1.5. Machine learning

The data pipeline begins by reading machine_learning.csv and normalizing header names. Numeric columns are converted from strings, heated in DM? is mapped to a binary yes/no indicator, and missing additive components are filled with zeros. Samples containing PBS (mM) or HEPES (mM) are removed from the dataset, as there are too few samples present.

The current configuration disables pH and pyruvate (mM) effects from the model, as they were about constant throughout the experiments. Fixed optimization values force heated in DM? and trityl (mM) to 0, as many of the DNP samples have been measured only in optimized conditions the model had difficulties noticing the detrimental effects.

Derived interaction features (D_2O * O_2 and D_2O * Tris) are computed explicitly and included in the model.

A GradientBoostingRegressor is trained separately for each target (low field, medium field, high field T_1). The GB model uses 300 estimators, maximum depth 3, learning rate 0.05, subsample 0.8, and random_state=42. Inputs are standardized with StandardScaler.

Model performance is evaluated by 5-fold cross-validated R^2. Feature importances are extracted from the GB model, and optional SHAP analysis is enabled to generate explainability plots and dependence curves.

Optimization is performed with scipy.optimize.differential_evolution using population size 20, maxiter=1000, tolerance $1e^{-6}$, mutation (0.5, 1.5), and recombination 0.9. During optimization, the constraint D_2O (%) = 100 - H_2O (%) is enforced along with fixed and binary feature constraints. Final optimal compositions and predicted T_1 values are saved to optimal_compositions.csv.

2. [1-^{13}C]pyruvate polarization losses during transfer

2.1. Magnetic field profile

Using the relaxation data obtained with the MFC, we calculated the polarization losses during transfer from the pyruvate polarizer to the measurement site. To do this, we measured the magnetic field profile along the transfer path. In our case, it was from the dDNP to the NMR system. The exact timing of the sample transfer – including dissolution, distribution into a smaller tube, actual transport, and insertion into the NMR spectrometer – was estimated, and the total duration was set to 19.5 s, as in our typical experiments. This yielded the magnetic field profile experienced by the sample during the transfer, shown in **Fig. S1** (reproduced from Ref. [2]). Based on this profile, the polarization loss (from an initial value set to 100%) was calculated for three different scenarios: (i) no additional magnets, (ii) transfer within a dedicated magnet between the dDNP and NMR systems, and (iii) a dissolution receiver vessel equipped with an additional magnet.

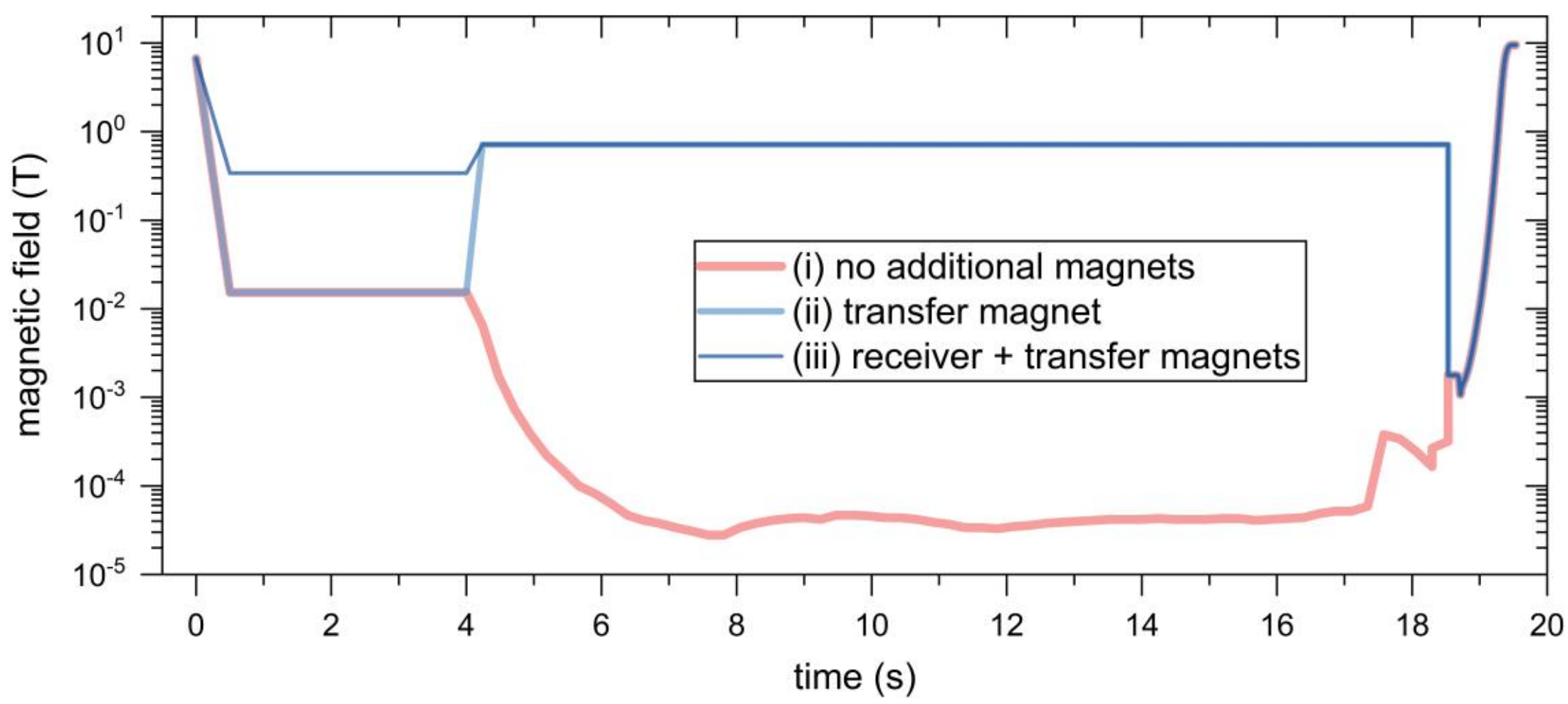

Fig. S1: Magnetic field profile and relaxation between the dDNP and the 9.4 T measurement site. *The three field profiles during transfer from the polarizer to the NMR have been measured to estimate the polarization losses as a result of sample transfer. Three cases have been considered: (i) no additional magnets , (ii) transfer within a dedicated magnet between the dDNP and NMR systems, and (iii) a dissolution receiver vessel equipped with an additional magnet. Figure reproduced from Ref.* [2].

2.2. NMRD fitting

The fitting was conducted using a model that included two inner sphere equations (R_{IS}) and chemical shift anisotropy (CSA) induced relaxation (R_{CSA}, Eq. 4 in the ESI of Ref. [3]).

The functions for R_{IS} was

$$R_{\mathrm{IS}} = \frac{(\Delta\omega)^2\,\tau_{\mathrm{C}}}{1+\left(\omega_{^{13}\mathrm{C}}\,\tau_{\mathrm{C}}\right)^2}, \qquad \text{Eq. 1}$$

where $\Delta\omega$ is a fitted coupling constant (not scalar in the traditional sense), S_X is the spin number of the coupled nucleus, τ_C is the correlation time characteristic of this interaction, $\omega_{^{13}C}$ is the Larmor angular frequencies of the coupled ^{13}C nucleus.

The function for R_{CSA} was

$$R_{CSA} = \frac{1}{5}\left(\omega_{^{13}C}\delta_{CSA}\right)^2 \frac{\tau_{CSA}}{1+\left(\omega_{^{13}C}\tau_{CSA}\right)^2}, \quad \text{Eq. 2}$$

where δ_{CSA} is the CSA of the studied nucleus and τ_{CSA} corresponding correlation time.

The NMRD had two elbows at fields below 1 T, which results in the necessity of having two functions like R_{IS}. The final fitting curve was achieved by fitting the following superposition:

$$R = R_{IS\_1} + R_{IS\_2} + R_{CSA}. \quad \text{Eq. 3}$$

Possibly, the two $\Delta\omega$ are the consequence of proton exchange at the COOH group and conversion between ketone and geminal diol (hydrate) form.

2.3. Relaxation of hyperpolarization

To estimate the remaining hyperpolarization, we solved the Bloch equation without thermal magnetization, assuming it is much less than the observed polarization value and SNR:

$$\frac{P(T)}{P(0)} = \exp\left(-\int_0^T \frac{dt}{T_1(B(t))}\right) = \exp\left(-R_1^{\mathrm{avg}}T\right) \quad \text{Eq 5}$$

Where T is the total time of the sample transfer, $T_1(B(t))$ is the relaxation time as a function of magnetic field, which depends on time and R_1^{avg} is the average T_1 relaxation rate constant experienced by the sample during the transfer.

2.4. Retained polarization after transfer

Using NMRDs, we estimated polarization losses during t_{pt} showcasing the gain in residual HP of [1-^{13}C]pyruvate (**Fig. S2**). The higher the retained hyperpolarization, the more sensitive hyperpolarized MRI is and the more time is available for the reliable quality control that improves the capabilities of in vitro and in vivo HP studies.

A clear difference between the no-magnet and transfer-magnet cases can be observed, with differences as significant as 29% more retained polarization for the trityl sample S6. The difference between cases (ii) and (iii) was less significant for pyruvate. However, it may provide a bigger relevance for quickly relaxing molecules such as [1-^{15}N]nicotinamide[4,5].

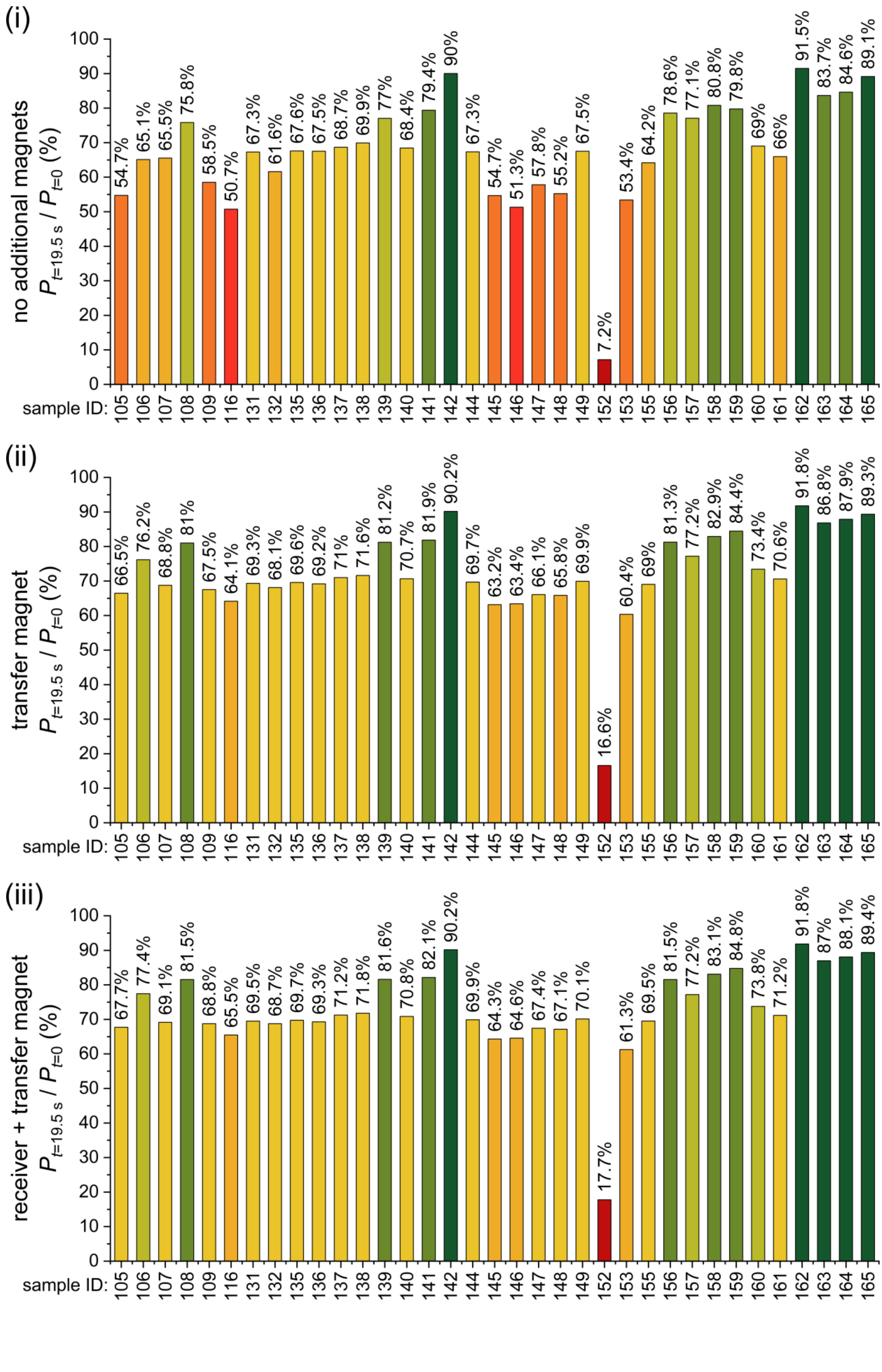
(i)
no additional magnets
$P_{t=19.5\,s}$ / $P_{t=0}$ (%)
0 10 20 30 40 50 60 70 80 90 100
54.7% 65.1% 65.5% 75.8% 58.5% 50.7% 67.3% 61.6% 67.6% 67.5% 68.7% 69.9% 77% 68.4% 79.4% 90% 67.3% 54.7% 51.3% 57.8% 55.2% 67.5% 7.2% 53.4% 64.2% 78.6% 77.1% 80.8% 79.8% 69% 66% 91.5% 83.7% 84.6% 89.1%
sample ID: 105 106 107 108 109 116 131 132 135 136 137 138 139 140 141 142 144 145 146 147 148 149 152 153 155 156 157 158 159 160 161 162 163 164 165
(ii)
transfer magnet
$P_{t=19.5\,s}$ / $P_{t=0}$ (%)
0 10 20 30 40 50 60 70 80 90 100
66.5% 76.2% 68.8% 81% 67.5% 64.1% 69.3% 68.1% 69.6% 69.2% 71% 71.6% 81.2% 70.7% 81.9% 90.2% 69.7% 63.2% 63.4% 66.1% 65.8% 69.9% 16.6% 60.4% 69% 81.3% 77.2% 82.9% 84.4% 73.4% 70.6% 91.8% 86.8% 87.9% 89.3%
sample ID: 105 106 107 108 109 116 131 132 135 136 137 138 139 140 141 142 144 145 146 147 148 149 152 153 155 156 157 158 159 160 161 162 163 164 165
(iii)
receiver + transfer magnet
$P_{t=19.5\,s}$ / $P_{t=0}$ (%)
0 10 20 30 40 50 60 70 80 90 100
67.7% 77.4% 69.1% 81.5% 68.8% 65.5% 69.5% 68.7% 69.7% 69.3% 71.2% 71.8% 81.6% 70.8% 82.1% 90.2% 69.9% 64.3% 64.6% 67.4% 67.1% 70.1% 17.7% 61.3% 69.5% 81.5% 77.2% 83.1% 84.8% 73.8% 71.2% 91.8% 87% 88.1% 89.4%
sample ID: 105 106 107 108 109 116 131 132 135 136 137 138 139 140 141 142 144 145 146 147 148 149 152 153 155 156 157 158 159 160 161 162 163 164 165

Fig. S2: Comparison of residual polarization of [1-^{13}C]pyruvate following a 19.5 s transfer from the polarizer to the measurement site without transfer magnet. *The field profile from Ref. [2] was used to estimate the losses of a normalized initial polarization of 100% through low fields during transfer without an additional field from a transfer magnet. The lowest residual polarization is expected for the DNP sample containing 4.5 mM Tris buffer and the highest for the degassed, deuterated pyruvate sample in 100% D_2O. The mapping from sample ID to sample composition can be found in* ***Tab. S1*** *or in the supplementary excel file (all_samples.xlsx).*

3. References

(1) Morris, G. A.; Freeman, R. Enhancement of Nuclear Magnetic Resonance Signals by Polarization Transfer. *J. Am. Chem. Soc.* **1979**, *101* (3), 760–762. https://doi.org/10.1021/ja00497a058.

(2) Peters, J. P.; Assaf, C. D.; Hövener, J.-B.; Pravdivtsev, A. N. Compact Magnetic Field Cycling System with the Range from nT to 9.4 T Exemplified with 13C Relaxation Dispersion and SABRE-SHEATH Hyperpolarization. arXiv June 10, 2025. https://doi.org/10.48550/arXiv.2506.08711.

(3) Kharkov, B.; Duan, X.; Rantaharju, J.; Sabba, M.; Levitt, M. H.; Canary, J. W.; Jerschow, A. Weak Nuclear Spin Singlet Relaxation Mechanisms Revealed by Experiment and Computation. *Phys. Chem. Chem. Phys.* **2022**, *24* (12), 7531–7538. https://doi.org/10.1039/D1CP05537B.

(4) Peters, J. P.; Brahms, A.; Janicaud, V.; Anikeeva, M.; Peschke, E.; Ellermann, F.; Ferrari, A.; Hellmold, D.; Held-Feindt, J.; Kim, N.; Meiser, J.; Aden, K.; Herges, R.; Hövener, J.-B.; Pravdivtsev, A. N. Nitrogen-15 Dynamic Nuclear Polarization of Nicotinamide Derivatives in Biocompatible Solutions. *Sci. Adv.* **2023**, *9* (34), eadd3643. https://doi.org/10.1126/sciadv.add3643.

(5) Peters, J.; Assaf, C.; Brahms, A.; Them, K.; Gerdsen, M.; Herges, R.; Hövener, J.-B.; Pravdivtsev, A. N. Chemically Induced Deceleration of Nuclear Spin Relaxation (CIDER) Preserves Hyperpolarization. *Sci. Adv.* **2025**, *11* (37), eadx2316. https://doi.org/10.1126/sciadv.adx2316.
